# A disease-specific patient reported outcome instrument for spine trauma is developed, validated and available! Re: Andrzejowski et al. Measuring functional outcomes in major trauma: can we do better?

**DOI:** 10.1007/s00068-022-02167-8

**Published:** 2022-11-15

**Authors:** Said Sadiqi, F. Cumhur Oner

**Affiliations:** Department of Orthopaedics, University Medical Center Utrecht, HP G05.228, P.O. Box 85500, 3508GA Utrecht, The Netherlands

Dear Editor,

We would like to congratulate the authors on the publication of the literature review showing the heterogeneity of patient reported outcome measures (PROMs) used in trauma patients [1].

The authors indicate that currently in the UK, the Trauma Audit Research Network (TARN) collects data up to 6 months. We believe this would in general be considered as rather a short-term follow-up period. In the discussion, a follow-up of at least 2 years (and up to 10 years) is recommended, which we would also favor to be able to evaluate long-term follow-up results. Also, it was interesting to read that the TARN database only gathered Euroqol 5-Dimension (EQ-5D) and Glasgow Outcome Score Extended (GOSE). It can be questioned whether these are appropriate to measure the outcomes of the trauma patient populations adequately. We agree with the authors that it is important to measure the quality of life (QoL) and functional outcomes adequately.

We were pleased to read that in this perspective the authors indicate the importance and comprehensiveness of the International Classification of Functioning, Disability and Health (ICF) system of the World Health Organization. On the other hand, we were very surprised that the AO Spine PROST (Patient Reported Outcome Spine Trauma) was not found in their literature search, thus, also not mentioned in the article. The AO Spine PROST is a disease-specific PROM for spine trauma patients, which was developed by the AO Spine Knowledge Forum Trauma following a multi-phase process. The systematic approach and methodology of the ICF were used as the basis for the development of the tool. This ICF classification system consists of more than 1400 categories to describe and classify individuals’ functioning, disability, and health. In the preparatory phase of the project, four different studies were completed. Three studies aimed to identify relevant ICF categories from different perspectives: research, [2] expert, [3] and patient perspective [4]. A fourth study investigated various response scales for their potential use in the tool [5]. In the next phase, out of 159 identified relevant ICF categories, 25 were selected as core categories during an international consensus conference [6]. Subsequently, the AO Spine PROST was developed by clustering those 25 core ICF categories into 19 items and implementing the items into the selected response scale [7].

It is important to realize that the main focus of the available measurement instruments used among spine trauma patients concerns pain. This also applies to the suggested PROMs by the authors: the NDI (Neck Disability Index), ODI (Oswestry Disability Index), or TDI (Total Disability Index). However, pain seems not to be the main issue in the recovery of spine trauma patients, rather functional impairments [4]. Therefore, the AO Spine PROST aims to measure the functional level and health status specifically after the traumatic event. This is reflected by the unique approach of the tool with a scale that ranges from 0 to 100, in which 0 indicates no function at all, and 100 the pre-injury level of function and health. The tool consists of a total of 19 questions that capture a broad range of aspects of functioning, such as walking and household activities, but also social life, emotional function, urinating, and bowel movement.

To the best of our knowledge, currently the AO Spine PROST has been, or is being, translated into 17 languages: Arabic, Dutch, English, Filipino, French, German, Hindi, Mandarin Chinese, Nepali, Norwegian, Portuguese, Romanian, Slovak, Spanish, Swahili, Thai, and Turkish. Out of these, the validation of 3 languages have been published so far: Dutch, English, and Nepali [8–10].

The AO Spine PROST has been developed for measuring outcomes among spine trauma patients. However, we believe it could also serve as an adequate basis for the development among other trauma patient populations. Thus, in response to the authors’ question: yes, we can do better. We would encourage colleagues around the world to investigate the applicability and validity of the AO Spine PROST among other specific trauma patient populations. Together with the many translations the tool has the potential to be useful in an international setting both for research and clinical purposes, and contribute to the improvement of the quality of health care in trauma patients.
