# Correction: A disease-specific patient reported outcome instrument for spine trauma is developed, validated and available! Re: Andrzejowski et al. Measuring functional outcomes in major trauma: can we do better?

**DOI:** 10.1007/s00068-023-02249-1

**Published:** 2023-03-09

**Authors:** Said Sadiqi, F. Cumhur Oner

**Affiliations:** Department of Orthopaedics, University Medical Center Utrecht, HP G05.228, P.O. Box 85500, 3508GA Utrecht, The Netherlands

**Correction: European Journal of Trauma and Emergency Surgery** 10.1007/s00068-022-02167-8

The article “A disease‑specific patient reported outcome instrument for spine trauma is developed, validated and available! Re: Andrzejowski et al. Measuring functional outcomes in major trauma: can we do better?”, written by Said Sadiqi, F. Cumhur Oner, was originally published electronically on the publisher’s internet portal on 15. November 2022 without open access. With the author(s)’ decision to opt for Open Choice the copyright of the article changed on 7. February 2023 to © The Authors 2022 and the article is forthwith distributed under a Creative Commons Attribution 4.0 International License, which permits use, sharing, adaptation, distribution and reproduction in any medium or format, as long as you give appropriate credit to the original author(s) and the source, provide a link to the Creative Commons licence, and indicate if changes were made. The images or other third party material in this article are included in the article's Creative Commons licence, unless indicated otherwise in a credit line to the material. If material is not included in the article's Creative Commons licence and your intended use is not permitted by statutory regulation or exceeds the permitted use, you will need to obtain permission directly from the copyright holder. To view a copy of this licence, visit http://creativecommons.org/licenses/by/4.0/.

